# Risk prediction models for hepatic encephalopathy following TIPS: a systematic review and meta-analysis

**DOI:** 10.3389/fmed.2026.1707035

**Published:** 2026-02-02

**Authors:** Ziji Fang, Zhenwei Liu, ZaiChun Pu, Qi Xia, Qin Zhang, Li Wang, Ping Jia, Xiaobin Tang

**Affiliations:** 1 School of Medicine, University of Electronic Science and Technology of China, Chengdu, China; 2 Department of Neurosurgery, Sichuan Provincial People's Hospital, University of Electronic Science and Technology of China, Chengdu, China; 3 Xindu District People's Hospital of Chengdu, Chengdu, China; 4 Department of PICC Nursing Center, Sichuan Provincial People's Hospital, University of Electronic Science and Technology of China, Chengdu, China; 5 Department of Day Surgery Ward, Sichuan Provincial People's Hospital, University of Electronic Science and Technology of China, Chengdu, China; 6 Department of Retired Personnel, Sichuan Provincial People’s Hospital, University of Electronic Science and Technology of China, Chengdu, China

**Keywords:** hepatic encephalopathy, predictive models, risk factors, systematic review/meta-analysis, transjugular intrahepatic portosystemic shunt

## Abstract

**Background and aims:**

Numerous risk-prediction models for hepatic encephalopathy (HE) after TIPS have been proposed, but their quality, discrimination, and clinical utility remain uncertain. We aimed to identify key predictors of post-TIPS HE and to critically appraise existing models.

**Methods:**

We performed a systematic review and meta-analysis of observational studies (inception 15 July 2025) from Chinese (VIP, Wanfang, CNKI, and CBM) and international (Embase, PubMed, Web of Science, and Cochrane Library) databases. Model quality and bias were assessed via PROBAST. Pooled AUCs and predictor effect sizes were calculated using STATA 15.0 and MedCalc.

**Results:**

A total of 24 studies (5,197 patients) yielded 32 unique models; the incidence of HE ranged from 19.9 to 46.6%. Discrimination was generally good (AUC range, 0.64–1.00; 30 models >0.70; 22 > 0.80). PROBAST flagged high bias—especially in model analysis. A meta-analysis produced a summary AUC of 0.815 (95%CI, 0.780–0.849). Consistent predictors (*p* < 0.01) included older age, diabetes, higher Child-Pugh score/class, elevated ammonia, and increased portal-to-splenic vein diameter ratio.

**Conclusion:**

Existing post-TIPS HE models demonstrate strong discrimination but suffer methodological limitations and bias. Future studies should employ multicenter cohorts, harmonized definitions, rigorous analytics, and external validation to yield robust, clinically actionable tools.

**Systematic review registration:**

https://www.crd.york.ac.uk/PROSPERO/view/CRD42024597699, CRD42024597699.

## 1 Introduction

Transjugular intrahepatic portosystemic shunt (TIPS) is an image-guided intervention that creates a low-resistance channel between the portal and hepatic veins to lower portal pressure in patients with portal hypertension-related liver disease (1). Originally introduced in the 1980s, TIPS has become a mainstay in tertiary centers for controlling variceal hemorrhage and refractory ascites (2, 3). Beyond hemostasis and fluid management, growing data indicate that TIPS confers long-term benefits, such as improved survival, fewer readmissions, and enhanced nutritional status and quality of life in advanced cirrhosis (4–7).

However, these gains must be balanced against the risk of hepatic encephalopathy (HE), the most frequent neurologic complication post-TIPS, which affects 23–54.5% of patients within 6 months (8, 9). HE—from mild cognitive decline to coma—drives morbidity, prolongs hospitalization, and elevates mortality (10, 11). Despite advances in peri-procedural care, post-TIPS HE remains unpredictable and may offset the procedure’s overall benefit.

Researchers have thus developed multivariable models—combining clinical parameters, imaging markers, and biochemical biomarkers—to stratify HE risk after TIPS. However, their methodological rigor, discrimination, and applicability across heterogeneous cohorts remain unexamined. We therefore performed a systematic review of post-TIPS HE prediction tools, critically appraising model development, validation methods, and key performance indices. By comparing their strengths and limitations, we provide hepatologists and interventional radiologists practical, evidence-based recommendations for integrating these tools into patient selection and peri-procedural management.

## 2 Materials and methods

This review adhered to the PRISMA 2020 statement (12), and its protocol was prospectively registered in PROSPERO (CRD42024597699).

### 2.1 Search strategy

We searched Chinese (VIP, Wanfang, CNKI, and CBM) and English (Embase, PubMed, Web of Science, and Cochrane Library) databases from inception to 15 July 2025 using MeSH and free-text terms for TIPS (“transjugular intrahepatic portosystemic shunt,” “portasystemic shunt,” and “transjugular intrahepatic”), HE (“hepatic encephalopathy” and “ammoniac encephalopathy”), and risk models (“nomogram,” “prediction model*,” and “risk assessment*”). Database-specific search syntax was applied, and reference lists of included studies and reviews were manually searched. Full search strategies are provided in the Supplementary materials.

We applied the PICOTS framework according to Checklist for Critical Appraisal and Data Extraction for Systematic Reviews of Prediction Modeling Studies (CHARMS) (13) to guide objective formulation, search strategy, and study selection (14).

P: Adults (≥18 years) undergoing TIPS for portal-hypertension complications.

I: Published risk-prediction models for post-TIPS HE.

C: Not applicable (no head-to-head model comparisons).

O: Development of hepatic encephalopathy.

T: Predictions based on baseline demographics, clinical scores, and laboratory data.

S: Models intended to yield individualized HE risk estimates to inform clinical decision-making and preventive measures.

### 2.2 Include inclusion and exclusion criteria

The inclusion criteria were as follows:

(1) Adults (≥18 years) undergoing TIPS; (2) studies developing HE risk-prediction models; (3) prospective or retrospective study design; and (4) published in English or Chinese.

The exclusion criteria were as follows:

(1) Duplicate publications; (2) models derived from systematic reviews or meta-analyses; (3) studies without full-text access or with insufficient abstract information; (4) studies from which relevant data could not be extracted; (5) conference abstracts, theses, and other unpublished materials; and (6) studies that analyzed risk factors without constructing predictive models.

### 2.3 Literature screening and data extraction

Study selection followed PRISMA guidelines. After de-duplication in Zotero, titles and abstracts were screened against our inclusion/exclusion criteria. Two reviewers then independently reviewed full texts for eligibility. Data were extracted into a CHARMS-based template and managed in Excel. Any discrepancies were resolved by discussion or, if required, by a third reviewer.

### 2.4 Quality evaluation of literature

Risk of bias and applicability were assessed using PROBAST (15). Bias was judged across four domains (participants, predictors, outcomes, and analysis) via 20 signaling questions: studies with all domains rated “low” were deemed low-risk, whereas any “high” or “unclear” rating conferred high overall risk. Applicability was evaluated in three domains (participants, predictors, and outcomes), with any “high” concern indicating poor applicability. Two independent reviewers performed all assessments; disagreements were resolved by discussion or, if needed, third-party adjudication.

### 2.5 Data synthesis and statistical analysis

Meta-analyses were performed in Stata 15.0, with between-study heterogeneity evaluated by the Q test and *I*^2^ statistic. A fixed-effects model was used when the *p*-value was >0.1 and *I*^2^ was <50%; otherwise, a random-effects model was applied. Predictors exhibiting substantial heterogeneity underwent sensitivity analyses to assess robustness and explore heterogeneity sources. Statistical significance was defined as a *p*-value of <0.05. Model discrimination was quantified by pooling AUCs in MedCalc; when only 95% CIs were reported, standard errors were derived by dividing the CI width by 3.92 (16).

## 3 Results

### 3.1 Study selection

**Figure 1:**
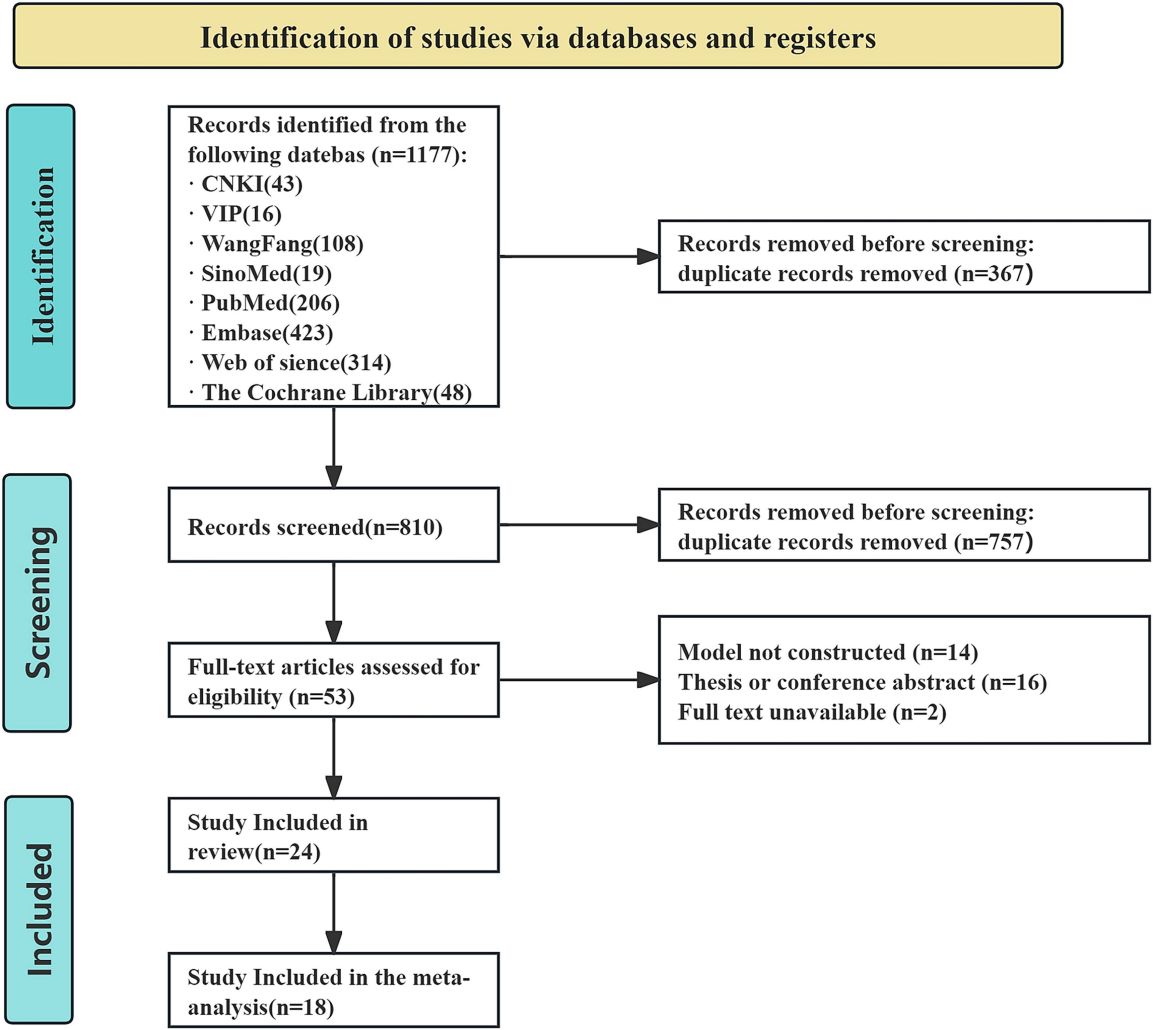
Flowchart depicting the systematic review’s literature search and selection based on PRISMA standards.

Figure 1 presents the PRISMA 2020 diagram, which outlines the detailed literature search methodology and outcomes.

The systematic search yielded 1,177 records; after removing 367 duplicates, 810 titles and abstracts were screened. Of these, 53 full texts were assessed for eligibility. Fourteen studies lacked predictive models, 16 were conference abstracts, and two were unavailable in full text. Ultimately, 24 studies satisfied all criteria and were included in the review.

### 3.2 Study characteristics

A total of 24 studies (16–39) were included (9 in Chinese, 15 in English), comprising 13 case–control, 7 retrospective cohort, and 1 prospective cohort design. Sample sizes ranged from 53 to 621 participants. Key characteristics are summarized in Table 1.

**Table 1:** Basic characteristics of studies included.

Author (year)	Country	Study design	Population	Follow-up time (month)	Sample size	Outcome	HE incidence
Li Yinglong* (2020) (17)	China	Retrospective study	1	1–39	262	HE	34.70%
Huang Tingping* (2022) (18)	China	Retrospective study	1	12	53	HE	26.40%
Li Xinyi* (2024) (19)	China	Retrospective study	1	6	113	HE	28.32%
Yong Liao (2023) (20)	China	Retrospective study	1	12	296	HE	19.93%
Yang Yang (2021) (16)	China	Retrospective study	1	12	185	HE	30.27%
Kejia Li (2024) (21)	China	Retrospective study	1	45.8	132	HE	28.03%
Yang Yang (2022) (22)	China	Retrospective study	1	12	191	HE	26.70%
Okan İnce (2023) (23)	The United States	Retrospective study	1	1	327	HE	32.11%
Sihang Cheng (2024) (24)	China	Retrospective study	1	12	130	HE	23.85%
Chongtu Yang (2022) (25)	China	Retrospective study	2	12	276	HE	22.10%
Sihang Cheng (2022) (26)	China	Retrospective study	1	None	106	HE	22.64%
Xiaochun Yin (2020) (27)	China	Retrospective study	1	12	373	HE	31.37%
Huan Tong (2021) (28)	China	Retrospective study	1	26	361	HE	20.22%
Xiaoqiong Chen (2024) (29)	China	Retrospective study	1	12	621	HE	30.27%
Yuan Wei* (2021) (30)	China	Retrospective study	1	3	108	HE	37.96%
Zhuo Songbo* (2023) (31)	China	Retrospective study	1	3	263	HE	22.05%
Yu Xiang* (2021) (32)	China	Retrospective study	2	12–70	106	HE	22.64%
Zhang Wenjing* (2024) (33)	China	Retrospective study	1	6	87	HE	31.03%
Wang Lanjing* (2024) (34)	China	Retrospective study	1	6	302	HE	21.52%
Silvia Nardelli (2016) (35)	Italy	Prospective study	2	6	82	HE	42.68%
Chuhan Wang (2023) (36)	China	Retrospective study	2	12	191	HE	46.60%
Chang Peng* (2025) (39)	China	Retrospective study	1	3	290	HE	29.31%
DeJia Liu (2025) (38)	China	Retrospective study	1	12	218	HE	15.1%
Gianvincenzo Sparacia (2025) (37)	Italy	Retrospective study	2	None	124	HE	30.6%

*Published in China; (1) Patients with liver cirrhosis and portal hypertension; (2) Patients with liver cirrhosis; HE, hepatic encephalopathy.

**Figure 2:**
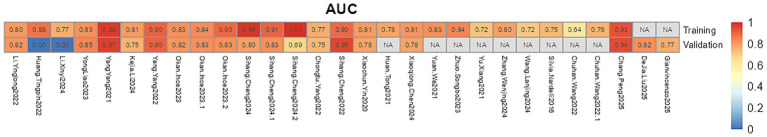
The AUC values of the prediction model.

Among 24 studies, AUC ranged from 0.64 to 1.00 (Figure 2). Except for one study, all retained continuous predictors, with variable selection by stepwise regression (*n* = 1), univariate → multivariate analysis (*n* = 16), or LASSO (*n* = 4). Missing data were variably addressed—multiple imputation without case count (*n* = 1), exclusion after reporting missingness (*n* = 1), or complete-case analysis (*n* = 10). Most models employed logistic regression (*n* = 16), with four using hybrid machine-learning approaches (e.g., random forest, SVM, and CatBoost), one using random forest alone, two using Cox models, and one using a competing-risks Cox model. Calibration was evaluated by both Hosmer–Lemeshow and calibration plots (*n* = 5), calibration plots alone (*n* = 7), while ten studies did not report any calibration assessment.

### 3.3 Models validation

Regarding validation, three studies performed both internal and external validation, twelve conducted internal validation only, and one reported external validation alone. The remaining studies did not perform any form of validation. Key model characteristics are summarized in Table 2.

**Table 2:** Basic features of the prediction models of the included studies.

Author (year)	Modeling methods	Variable screening method	Modeling methods	EPV	AUC (training/validation)	Model validation method	Model calibration method	Missing data processing method	Continuous data processing method	Model presentation
Li Yinglong* (2020) (17)	Classification model	Single-factor analysis	LR	30.333	0.805/0.816	Internal validation	Hosmer Lemeshow test, Calibration plot	Exclusion included	Continuous variable	Nomogram
Huang Tingping* (2022) (18)	Classification model	Single-factor analysis	LR, SVM, RF	4.667	0.770/−	–	Hosmer Lemeshow test, Calibration plot	–	Continuous variable	Nomogram
Li Xinyi* (2024) (19)	Classification model	Single-factor analysis	LR	8.000	0.875/−	Internal validation	–	–	Continuous variable	–
Yong Liao (2023) (20)	Classification model	Single-factor analysis	LR	11.800	0.828/0.846	Internal validation (Bootstrap)	Calibration plot	Exclusion included	Continuous variable	Nomogram
Yang Yang (2021) (16)	Classification model	Single-factor analysis	LR	14.000	0.978/0.969	Internal validation	Calibration plot	Exclusion included	Continuous variable	Nomogram
Kejia Li (2024) (21)	Prognostic model	Single-factor analysis	Cox regression	12.333	0.806/0.751	Internal validation	Calibration plot	-	Continuous variable	Nomogram
Yang Yang (2022) (22)	Classification model	Lasso regression, backward selection	LR	7.286	0.901/0.903	Internal validation	Hosmer Lemeshow test, Calibration plot	Exclusion included	Continuous variable	Nomogram
Okan İnce (2023) (23)	Classification model	Single-factor analysis	LR, SVM, CatBoost	15.000	SVM: 0.830/0.820 LR: 0.840/0.830 CATBOOST: 0.900/0.830	Internal validation (K-fold cross validation)	–	Exclusion included	Continuous variable	–
Sihang Cheng (2024) (24)	Classification model	Single-factor analysis, lasso regression	LR, SVM, RF	2.818	SVM: 0.990/0.800 LR: 0.910/0.830 RF: 1.000/0.690	External validation	–	Exclusion included	Continuous variable	–
Chongtu Yang (2022) (25)	Classification model	Lasso regression	LR	15.250	0.770/0.750	External verification	Hosmer Lemeshow test, Calibration plot, Brier score	Multiple interpolation	Continuous variable	Nomogram
Sihang Cheng (2022) (26)	Classification model	–	LR	6.000	0.899/0.955	Internal validation (K-fold cross validation)	–	–	Continuous variable	–
Xiaochun Yin (2020) (27)	Prognostic model	Single-factor analysis	Cox regression	23.400	0.809/0.783	Internal validation (Bootstrap)	Calibration plot	Exclusion included	Continuous variable	Nomogram
Huan Tong (2021) (28)	Classification model	Single-factor analysis	LR	12.167	0.783/−	Internal validation	–	censored-data analysis	Continuous variable	Formula of risk score
Xiaoqiong Chen (2024) (29)	Classification model	Lasso regression	LR	47.000	0.814/0.781	External validation	Calibration plot	Exclusion included	Continuous variable	Web calculator
Yuan Wei* (2021) (30)	Classification model	Single-factor analysis	LR	13.667	0.828/−	–	Calibration plot	–	Continuous variable	Formula of risk score
Zhuo Songbo* (2023) (31)	Classification model	Single-factor analysis	LR	11.600	0.841/−	–	–	–	Continuous variable	Formula of risk score
Yu Xiang* (2021) (32)	Classification model	Single-factor analysis	LR	12.000	0.723/−	–	–	–	Continuous variable	Formula of risk score
Zhang Wenjing* (2024) (33)	Classification model	Single-factor analysis	LR	13.500	0.800/−	–	–	Exclusion included	Continuous variable	Formula of risk score
Wang Lanjing* (2024) (34)	Classification model	Single-factor analysis	LR	16.250	0.716/−	–	–	–	Continuous variable	Nomogram
Silvia Nardelli (2016) (35)	Prognostic model	Single-factor analysis	Fine-Gray test	11.667	0.750/−	–	–	–	Continuous variable	Formula of risk score
Chuhan Wang (2022) (36)	Classification model	Single-factor analysis	LR	22.250	Model 1: 0.64/− Model 2: 0.76/−	–	Calibration plot	Exclusion included	Categorical variables	Nomogram
Chang Peng* (2025) (39)	Classification model	Lasso regression	LR	8.500	0.933/0.944	Internal validation	Hosmer Lemeshow test	–	Continuous variable	Nomogram
DeJia Liu (2025) (38)	Classification model	Single-factor analysis	LR, RF, XGBoost	11.000	−/0.825	Internal validation	Hosmer Lemeshow test, Calibration plot, Brier score	–	Continuous variable	SHAP
Gianvincenzo Sparacia (2025) (37)	Classification model	Principal component analysis	MLP, RF, SVC, DT	3.800	KNN: −/0.77	Internal validation	–	–	Continuous variable	–

*Published in China; –, Not reported in the literature; LR, Logistic regression; SVM, Support vector machine; RF, Random forest; MLP, Multilayer perceptron; KNN, K-nearest neighbor; SVC, Support vector classifier; DT, Decision tree.

### 3.4 Results of quality assessment

PROBAST was used to evaluate bias and applicability across the 24 studies (Table 3). (1) Participants: Four case–control studies (18, 21, 22, 26) were judged high risk; nested case–control and cohort designs were low risk. (2) Predictors: Four studies (16, 23, 28, 36) were high risk because not all predictors were available at model use; in twelve studies (17–19, 21, 22, 25, 26, 30, 31, 33, 34, 37) predictor definitions or timing were insufficiently reported, yielding unclear risk. (3) Outcome: Thirteen studies (17–19, 21–26, 28, 30, 32, 33) had unclear risk because it was not specified whether outcome assessment was blinded to predictors or the interval between predictor and outcome measurement. (4) Analysis: Ten studies (16, 17, 20, 22–24, 27–29, 33, 36) used complete-case analysis; one (25) study used multiple imputation and the remaining study did not report methods for handling missing data or adjusting for overfitting, resulting in unclear or high risk.

**Table 3:** The risk of bias and suitability evaluation.

Author (year)	Study type	ROB				Applicability			Overall	
		Participants	Predictors	Outcome	Analysis	Participants	Predictors	Outcome	ROB	Applicability
Li Yinglong* (2020) (17)	B	+	?	?	−	+	?	+	−	?
Huang Tingping* (2022) (18)	A	−	?	?	−	+	+	?	−	?
Li Xinyi* (2024) (19)	A	+	?	?	−	+	?	+	−	?
Yong Liao (2023) (20)	B	+	−	+	−	+	+	+	−	+
Yang Yang (2021) (16)	B	+	+	+	−	+	+	+	−	+
Kejia Li (2024) (21)	B	−	?	?	−	−	?	+	−	−
Yang Yang (2022) (22)	B	−	?	?	−	−	?	+	−	−
Okan İnce (2023) (23)	B	+	−	?	−	+	?	?	−	?
Sihang Cheng (2024) (24)	B	+	+	?	−	+	+	+	−	+
Chongtu Yang (2022) (25)	B	+	?	?	−	+	?	+	−	?
Sihang Cheng (2022) (26)	B	−	?	?	−	+	+	?	−	?
Xiaochun Yin (2020) (27)	B	+	+	+	−	+	+	+	−	+
Huan Tong (2021) (28)	A	+	−	?	−	+	?	+	−	?
Xiaoqiong Chen (2024) (29)	B	+	+	+	−	+	+	+	−	+
Yuan Wei* (2021) (30)	A	+	?	?	−	+	?	+	−	?
Zhuo Songbo* (2023) (31)	A	+	?	+	−	+	?	+	−	?
Yu Xiang* (2021) (32)	A	+	+	?	−	+	+	?	−	?
Zhang Wenjing* (2024) (33)	A	+	?	?	−	+	?	+	−	?
Wang Lanjing* (2024) (34)	A	+	?	+	−	+	?	+	−	?
Silvia Nardelli (2016) (35)	A	+	+	+	−	+	+	+	−	+
Chuhan Wang (2022) (36)	A	+	−	+	−	+	+	+	−	+
Chang Peng (2025) (39)	B	+	+	+	−	+	+	+	−	+
DeJia Liu (2025) (38)	B	+	+	+	?	+	+	+	−	+
Gianvincenzo Sparacia (2025) (37)	B	+	?	+	−	+	+	+	−	+

*Published in China; ROB, risk of bias; A, development only; B, development and validation in the same publication; + indicates low ROB/low concern regarding applicability; – indicates high ROB/high concern regarding application; ? indicates unclear ROB/unclear concern regarding applicability.

## 4 Meta-analysis of validation models included in the review

**Figure 3:**
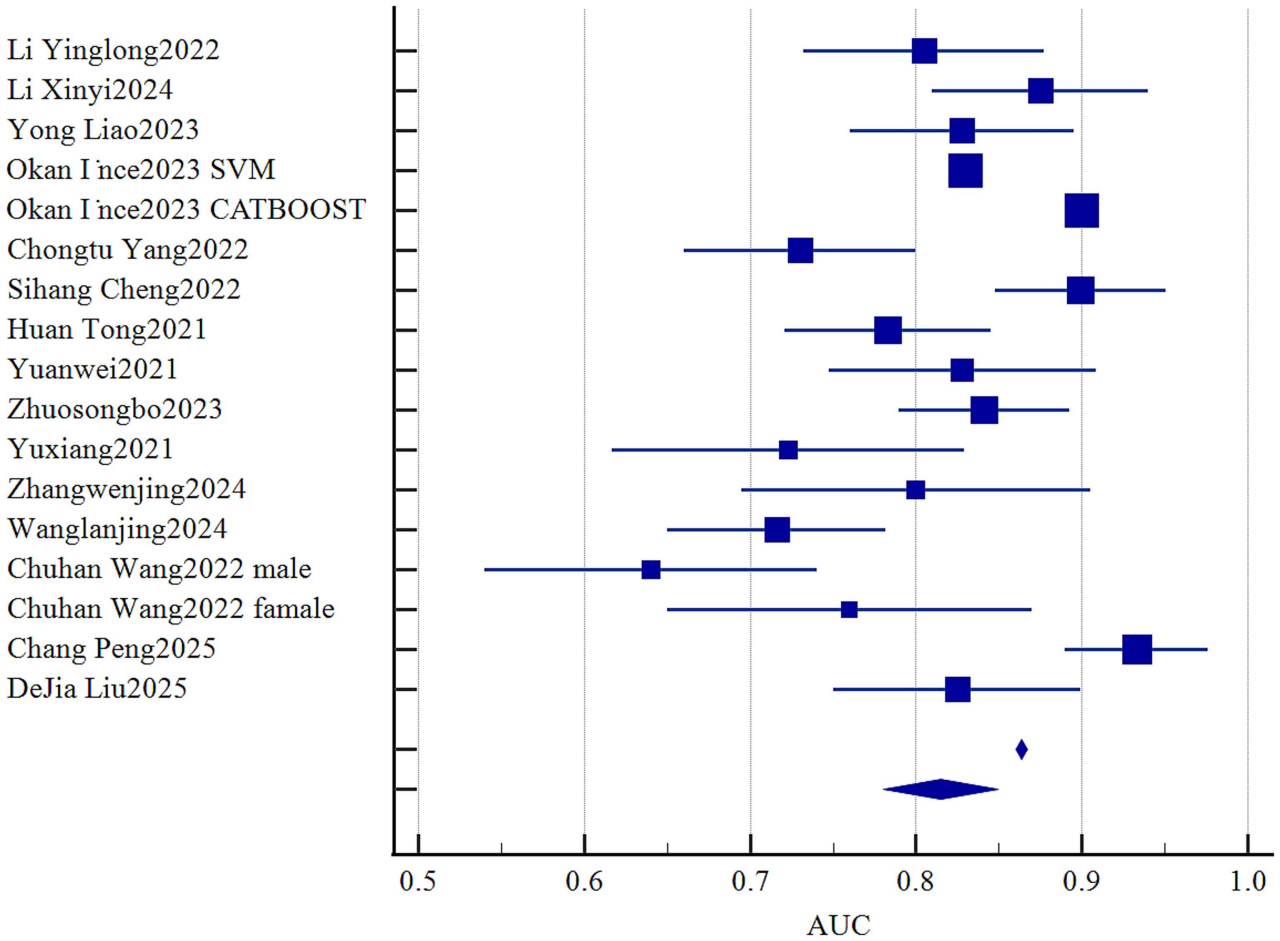
Forest plot of the pooled AUC values.

**Figure 4:**
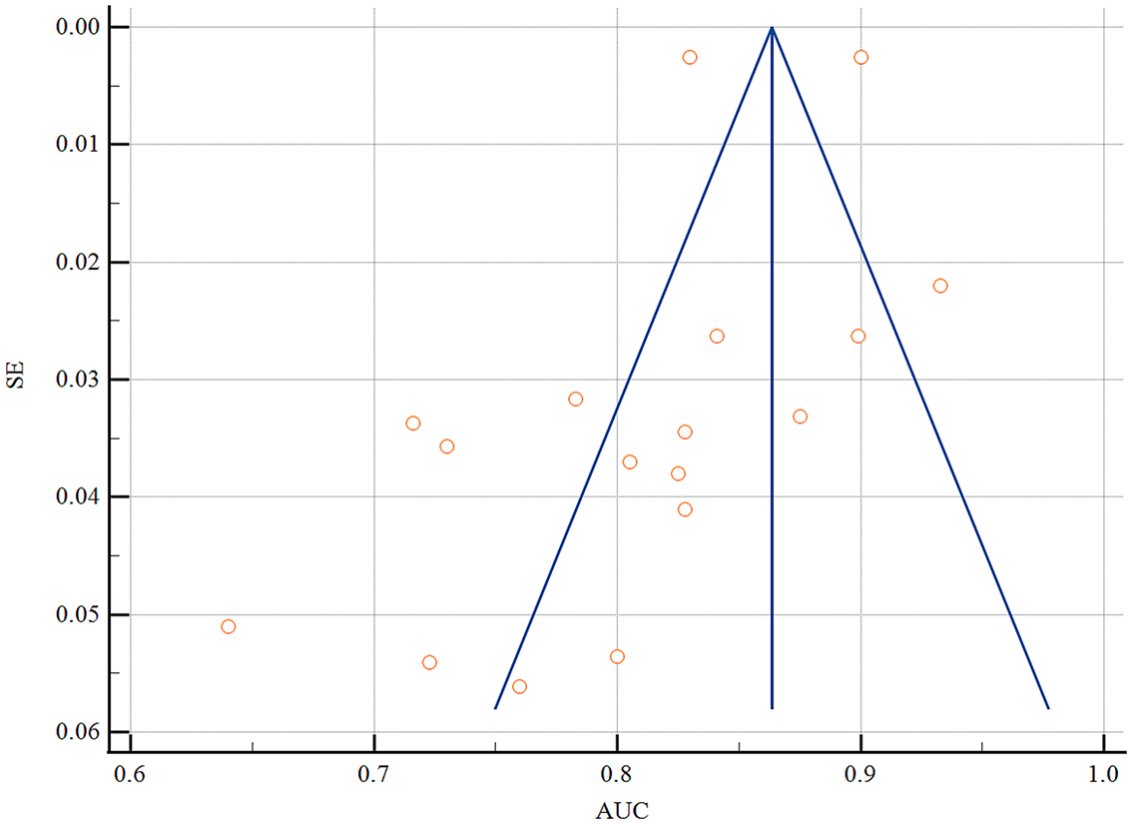
Funnel plot of the pooled AUC values.

Statistical pooling of model discrimination (AUC) was performed in MedCalc (Figure 3). This summary analysis primarily utilizes the AUC metric from the model development dataset. Given high heterogeneity (*I*^2^ = 96.56%, *p* < 0.001), a random-effects model yielded a summary AUC of 0.815 (95%CI, 0.780–0.849), reflecting strong overall discrimination. Publication bias was negligible, as evidenced by a symmetrical funnel plot (Figure 4) and a non-significant Egger’s test (*p* = 0.308). Subgroup and meta-regression analyses stratified by modeling algorithm (logistic regression vs. machine learning), validation type (internal vs. external), events-per-variable (≥10 vs. <10), and missing-data strategy (exclusion vs. imputation) failed to explain the remaining heterogeneity (*I*^2^ > 85%), suggesting unmeasured clinical or methodological factors.

**Figure 5:**
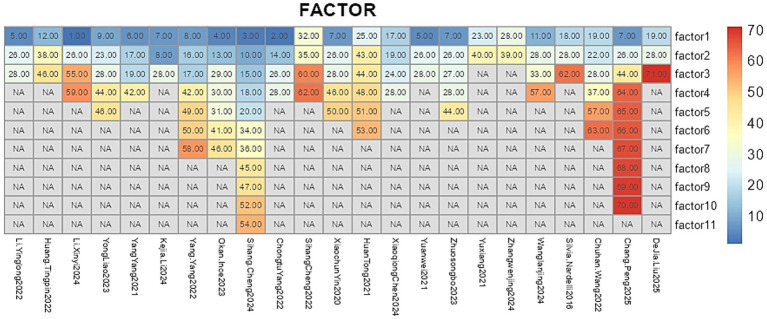
Predictors of the prediction model. In this study, variable codes are defined as follows: 1, denotes preoperative main portal vein diameter; 2, postoperative portosystemic pressure gradient (PPG); 3, Percentile 25-2; 4, operating surgeon; 5, fibrinogen; 6, direct bilirubin; 7, diabetes mellitus; 8, ascites; 9, indocyanine green retention at 15 min (ICG-R15); 10, Median intensity-2; 11, international normalized ratio (INR); 12, triglycerides; 13, indirect bilirubin; 14, sarcopenia; 15, Min intensity-2; 16, ratio of liver transverse diameter to fissure diameter; 17, PSR; 18, Percentile 15-2; 19, Child–Pugh score; 20, Percentile 30-2; 21, fibrinogen (duplicate); 22, visceral fat area index <53.52; 23, percentage decrease in PPG; 24, maximum portal vein diameter; 25, MELD score ≥10; 26, Child–Pugh class; 27, history of hepatitis B; 28, age; 29, MELD-Na score; 30, surgical complications; 31, portal vein puncture site; 32, Correlation_angle90_offset4; 33, white blood cell-to-platelet ratio (WBC/PLT); 34, Percentile 20-2; 35, RunLengthNonuniformity_angle0_offset4; 36, Percentile 40-2; 37, subcutaneous fat area index <70.05; 38, probiotic use; 39, albumin–bilirubin (ALBI) score; 40, high-sensitivity C-reactive protein (hs-CRP); 41, pre-TIPS esophagogastric variceal bleeding; 42, maximum hepatic fissure diameter; 43, alcoholic cirrhosis; 44, blood ammonia; 45, InverseDifferenceMoment_AllDirection_offset4_SD-1; 46, serum creatinine; 47, MeanValue-2; 48, ≥60% reduction in portal pressure; 49, anteroposterior diameter ratio of left to right liver lobe; 50, serum sodium; 51, pleural effusion; 52, Percentile 10-2; 53, stent diameter; 54, C-reactive protein (CRP); 55, Freiburg Index of Post-TIPS Survival (FIPS) score; 56, gamma-glutamyl transpeptidase (GGT); 57, prealbumin; 58, maximum anteroposterior diameter of the right liver lobe; 59, gamma-glutamyl transferase (GGT); 60, Inertia_AllDirection_offset4_SD; 61, RunLengthNonuniformity_AllDirection_offset7_SD; 62, cholinesterase (CHE); and 63, height; 64, total bilirubin; 65, Prothrombin time; 66, IL-6; 67, IL-18; 68, glial fibrillary acidic protein; 69, postoperative gastrointestinal dysfunction; 70, monocyte chemoattractant protein-1; 71, portal vein thrombosis.

Across studies, 71 candidate predictors emerged (Figure 5), with age, diabetes history, Child-Pugh score/class, blood ammonia, and portal–splenic vein diameter ratio—each examined in ≥2 cohorts—significantly associated with post-TIPS HE risk (Table 4). Sensitivity analyses confirmed the robustness of these associations despite heterogeneity.

**Table 4:** The results of the meta-analysis of the predictors.

Predictor	Included studies (*n*)	Heterogeneity test		The effect model	Meta	
		*I*^2^ (%)	*p* value		OR/HR (95%CI)	*p* value
Age (16, 22, 24, 28, 30, 32–34, 36–39)	9^1^	8.2	0.367	Fixed	1.03 (1.02, 1.04)	**<0.01**
	3^2^	23.1	0.272	Fixed	1.04 (1.02, 1.05)	**<0.01**
Diabetes (21, 27, 31, 39)	2^1^	0	0.638	Fixed	2.089 (1.45,3.02)	**<0.01**
	2^2^	0	0.347	Fixed	2.158 (1.38,3.39)	**<0.01**
Child-Pugh score (23, 32, 39)	3^1^	59.5	0.040	Random	1.77 (1.53, 2.04)	**<0.01**
Child-Pugh class (16, 21, 28, 30, 33, 34)	6^1^	8.0	0.337	Fixed	2.26 (1.66, 3.09)	**<0.01**
Blood ammonia (22, 31, 34)	3^1^	59	0.063	Random	1.01 (1.01,1.02)	**<0.01**
Albumin (37, 39)	2^1^	66.9	0.082	Random	0.99 (0.98, 0.99)	0.10
Fibrae sanguis (16, 33)	2^1^	90.1	0.002	Random	0.81^1^ (0.76, 0.86)	0.24
The diameter ratio of portal vein to splenic vein (23, 25, 32)	3^1^	65.8	0.054	Random	3.79 (2.56, 5.61)	**<0.01**
Maximum diameter of liver laceration (22, 25)	2^1^	93.7	<0.01	Random	1.21 (0.99, 1.48)	0.317

1 is OR value; 2 is HR value.

The bold formatting is used solely to highlight these significant variables and carries no intrinsic meaning.

## 5 Discussion

In 24 studies (35 predictive models, 5,197 patients), the reported incidence of HE after TIPS ranged from 19.9 to 46.6%. Since 2016, many models have shown encouraging discriminative performance (30 models with AUC > 0.70, 22 with AUC > 0.80), but substantial methodological heterogeneity undermines confidence in their clinical applicability. Most models were developed retrospectively and relied primarily on internal validation, with only five studies reporting external validation—features that increase the risk of selection and information bias and limit generalizability. Predictor selection frequently relied on univariable screening, a practice that can overlook important multivariable associations and fails to address multicollinearity or interaction effects (40). Handling of missing data was inconsistent: only one study used multiple imputation, ten excluded incomplete cases, and the majority did not report a clear strategy, further weakening the robustness and reproducibility of results.

Despite these limitations, three recurring predictor domains emerged: *patient-specific factors* (e.g., age and history of diabetes), *preoperative clinical indicators* (e.g., Child-Pugh score and blood ammonia level), and *imaging-based parameters*.

Advanced age is associated with an increased risk of HE, likely mediated through age-related gastrointestinal dysmotility, compositional shifts in the gut microbiota, and diminished extrahepatic ammonia detoxification capacity (41, 42). Physiologically, TIPS further augments systemic exposure to gut-derived ammonia by circumventing hepatic sinusoidal clearance (43, 44). Similarly, diabetes predisposes patients to HE through multiple mechanisms, such as insulin resistance-induced impairment of hepatic and skeletal muscle ammonia metabolism (45), and chronic hyperglycemia-driven upregulation of intestinal glutaminase activity and permeability, which together facilitate ammonia and endotoxin translocation (46, 47); post-TIPS diversion of portal flow further compromises first-pass hepatic extraction, thereby amplifying this risk (48, 49).

Higher pre-TIPS liver dysfunction scores reflect reduced synthetic function and intrinsic shunting, which TIPS may exacerbate (50). Although preprocedural ammonia level was prognostic in several reports, assay variability and lack of standardization limit its routine use (51); standardized protocols or point-of-care assays could improve its utility. Emerging biochemical markers (e.g., glutamine/glutamate ratios and phenylacetate) and dynamic flow metrics (Doppler or 4D-flow MRI) may enhance risk stratification beyond static measures and merit prospective evaluation.

Recent studies have increasingly indicated that malnutrition and sarcopenia are modifiable risk factors for hepatic encephalopathy (HE) after TIPS. Skeletal muscle plays a critical role in extrahepatic ammonia metabolism, and a reduction in muscle mass can markedly impair peripheral ammonia clearance, thereby increasing susceptibility to HE. In our dataset, several models included nutrition- or body composition-related variables—such as CT- or BIA-derived measures of muscle mass or fat distribution, prealbumin levels, or low-fat mass indices (reported in the variable lists as “sarcopenia,” “prealbumin,” or “visceral fat area index”). However, due to substantial heterogeneity in measurement methods, cutoff values, and reporting strategies, these variables did not demonstrate consistent stability in pooled analyses. In other words, while current evidence supports the biological plausibility and potential clinical relevance of nutrition-related indicators, methodological heterogeneity (such as assessment tools, thresholds, and follow-up time points) has hindered their consistent confirmation across prediction models. Therefore, we recommend that future model development prioritize internationally accepted definitions of sarcopenia (e.g., EWGSOP2 or AWGS) and report raw imaging-based measurements to facilitate cross-cohort comparisons and individual participant data (IPD) meta-analyses.

## 6 Conclusion

Although many existing models demonstrate promising discriminative performance, the substantial methodological heterogeneity across studies—particularly regarding modeling frameworks, predictor selection, and validation strategies—undermines the reliability and generalizability of current evidence. AUCs derived from various modeling frameworks are often highly heterogeneous and emphasize different aspects of predictive performance; therefore, simply pooling these AUCs into a single summary metric is methodologically questionable and may misrepresent a model’s clinical utility. To move the field forward, future studies should adopt prospective, multicenter designs with clearly pre-specified predictor sets, apply modern multivariable selection techniques (e.g., LASSO/ridge) (52, 53) and routine multiple imputation for missing data (54), and perform transparent reporting and external validation in line with TRIPOD and PROBAST (55). Notably, prognostic and diagnostic modeling frameworks should be explicitly separated, and pooled estimates (such as aggregated AUCs) should not be interpreted uncritically when they combine fundamentally different model types (e.g., logistic, Cox) since such pooling can be misleading.

### 6.1 Limitation

This systematic review has several limitations that should be carefully considered when interpreting the findings. First, approximately two-thirds of the included studies were derived from East Asian cohorts, predominantly from mainland China. This geographic concentration may limit the generalizability of the results to other regions, as differences in etiological profiles (e.g., proportions of viral versus alcohol-related liver disease), TIPS indications, and peri-procedural management strategies (such as perioperative antibiotic use, shunt design, and nursing practices) may influence both the incidence of HE and the external validity of identified predictors. Nevertheless, it is noteworthy that earlier cohort studies from Europe and North America have also reported several core predictors, suggesting that certain factors—such as age, Child-Pugh score, and blood ammonia levels—exhibit consistency across regions. However, these observations still require confirmation through prospective, multicenter, multinational studies. To enhance transparency, we have listed the geographic origin of each included study in the supplementary materials to allow readers to better assess the applicability of our findings.

Second, several potentially important predictors, such as a history of hepatic encephalopathy and nutritional status or sarcopenia, were inconsistently reported or variably defined across the original studies, resulting in a lack of consistent support in pooled model analyses. With respect to prior HE, some studies excluded patients with overt cognitive impairment or recurrent HE episodes, while others did not explicitly document mild or covert cognitive dysfunction at baseline. In addition, a history of HE often coexists with more advanced liver dysfunction and malnutrition, leading to strong collinearity with liver function scores and nutritional variables and increasing the likelihood of exclusion during multivariable model selection. Consequently, although earlier studies have suggested an association between prior HE or baseline cognitive impairment and post-TIPS HE risk, this variable did not emerge as a consistently significant predictor in our pooled analysis due to heterogeneity in definitions, sampling strategies, and statistical approaches. Future studies should incorporate systematic documentation of prior HE and standardized assessments of mild cognitive impairment to better delineate their independent predictive value.

Third, malnutrition and sarcopenia have gained increasing attention in recent years as biologically plausible mechanisms contributing to HE. Our list of candidate predictors included several nutrition- and body composition-related variables, such as sarcopenia, subcutaneous or visceral fat indices, and prealbumin levels. However, substantial variability in measurement methods (e.g., CT-based cross-sectional assessments, body mass index-based thresholds, or laboratory surrogate markers) and cutoff values across studies resulted in unstable findings in pooled analyses. Although existing evidence supports the role of muscle and nutritional depletion in increasing HE susceptibility—primarily through reduced peripheral ammonia clearance—methodological heterogeneity limits comparability. We therefore recommend that future models incorporate standardized sarcopenia assessments and nutritional scoring systems at the design stage and report detailed measurement methods and thresholds to facilitate subsequent synthesis and comparison.

Finally, methodological limitations should also be acknowledged. Most included studies were retrospective in nature, and only a minority performed external validation. In addition, there was no uniform definition of HE or standardized follow-up window (e.g., mixing HE incidence within 6- and 12-month periods), and approaches to handling missing data varied considerably, with complete-case analysis being the most common. These factors are likely to increase heterogeneity and may introduce bias in the estimation of predictive performance. To address this issue, we have provided a comprehensive summary table in the supplementary materials detailing the number of events, events per variable (EPV), missing data handling strategies, and external validation status for each model, enabling readers to better evaluate their robustness and potential clinical applicability.

## Data Availability

The original contributions presented in the study are included in the article/supplementary material, further inquiries can be directed to the corresponding authors.
